# Vitamin D Receptor Expression in Dogs

**DOI:** 10.1111/jvim.15052

**Published:** 2018-02-22

**Authors:** J.A. Cartwright, A.G. Gow, E. Milne, D. Drummond, S. Smith, I. Handel, R.J. Mellanby

**Affiliations:** ^1^ Division of Veterinary Clinical Studies, Royal (Dick) School of Veterinary Studies and The Roslin Institute Hospital for Small Animals, Easter Bush Veterinary Centre, The University of Edinburgh Roslin United Kingdom

**Keywords:** Immunohistochemistry, Inflammatory bowel disease, Chronic enteropathy, Tight junctions

## Abstract

**Background:**

There is growing evidence linking low blood vitamin D concentration to numerous diseases in people and in dogs. Vitamin D influences cellular function by signaling through the vitamin D receptor (VDR). Little is known about which non‐skeletal tissues express the VDR or how inflammation influences its expression in the dog.

**Objectives:**

To define which non‐skeletal canine tissues express the VDR and to investigate expression in inflamed small intestine.

**Animals:**

Thirteen non‐skeletal tissues were collected prospectively from 6 control dogs. Thirty‐five dogs diagnosed with a chronic enteropathy (CE) and 24 control dogs were prospectively enrolled and duodenal biopsies were evaluated for VDR expression.

**Methods:**

Prospective; blinded assessment of canine intestinal VDR. Dogs with CE were included once other identifiable causes of intestinal disease were excluded. Age matched controls were included with no intestinal clinical signs. VDR expression was assessed immunohistochemically in all samples, using a Rat IgG VDR monoclonal antibody. Quantitative real‐time polymerase chain reaction (qPCR) was also used for duodenal biopsies.

**Results:**

VDR expression as assessed by immunohistochemistry (IHC) was highest in the kidney, duodenum, skin, ileum and spleen, and weak in the colon, heart, lymph node, liver, lung, and ovary. Gastric and testicular tissue did not express the VDR. There was no statistical difference in duodenal VDR expression between the 24 healthy dogs and 34 dogs with CE when quantified by either qPCR (*P* = 0.87) or IHC (*P* = 0.099).

**Conclusions and Clinical Importance:**

The lack of down regulation of VDR expression in inflamed intestine contrasts with previous studies in humans. Our findings support future studies to investigate whether vitamin D and its analogues can be used to modulate intestinal inflammation in the dog.

**Abbreviations**
25(OH)D: 25 hydroxyvitamin D
1,25(OH)2D: 1, 25 dihydroxyvitamin D
ACTB: actin beta
cDNA: complementary deoxyribonucleic acid
CE: chronic enteropathy
CIBDAI: canine inflammatory bowel disease activity index
CLD2: claudin 2
CYP24A1: 1,25‐dihydroxyvitamin D3 24‐hydroxylase
CYP27B1: 25‐hydroxyvitamin D3 1‐alpha‐hydroxylase
ECAD: E‐cadherin
GAPDH: glyceraldehyde 3‐phosphate dehydrogenase
IBD: inflammatory bowel disease
IgG: immunoglobulin G
IHC: immunohistochemistry
Log2RQ: log2 transformed relative quantification data
OS: overall score
PLE: protein losing enteropathy
PTH: parathyroid hormone
QPCR: quantitative real‐time polymerase chain reaction
REST: relative expression software tool
RNA: ribonucleic acid
RXR: retinoid X receptor
SDHA: succinate dehydrogenase complex subunit A
TBST: tris buffered saline tween
VDR: vitamin D receptor
WSAVA: World Small Animal Veterinary Association

Vitamin D exerts its metabolic effects largely through signaling via the vitamin D receptor (VDR). Numerous cell types express the VDR,^1^, ^2^, ^3^ a ligand activated transcription factor, which is specific for and activated by binding 1, 25 dihydroxyvitamin D (1,25(OH)_2_D) and other vitamin D metabolites. The cytoplasmic VDR then translocates to the nucleus before heterodimerization with a retinoid X receptor (RXR), after which the heterodimer binds to vitamin D response elements. Finally, this binding results in recruitment of various other nuclear proteins into a transcriptional complex.^4^ This complex initiates and regulates the rate of transcription of target genes by Ribonucleic acid (RNA)‐polymerase II.^5^ There are also several recognized non‐genomic actions of 1,25(OH)_2_D that are reported to be facilitated by a different receptor,^6^ such as elevation of cyclic guanosine monophosphate levels, activation of protein kinase C and increases in intracellular calcium levels.^7^

The binding of 1,25(OH)_2_D to the VDR can influence a wide range of biological effects including cellular differentiation, proliferation, and phenotypic changes.^8^, ^9^ While the traditional roles of vitamin D have focused on its role in the maintenance of skeletal health, the non‐skeletal roles of vitamin D have been intensively investigated over the last 30 years since the discovery that VDR was expressed by a wide range of cell types. For example, many studies have found an association between low serum concentrations of 25 hydroxyvitamin D (25(OH)D) and the development and progression of various diseases in humans.^10^, ^11^, ^12^ These include hypertension, diabetes, cancer, cardiovascular diseases, autoimmune conditions, and infectious diseases, as well as Crohn's disease and ulcerative colitis,^13^, ^14^ which are both forms of inflammatory bowel disease (IBD).

The non‐skeletal effects of vitamin D have only recently been investigated in companion animals.^15^ Serum 25(OH)D concentrations are lower in dogs with several chronic and inflammatory diseases including spirocercosis,^16^ congestive heart failure,^17^ renal disease,^18^ cancer,^19^ and dogs with a chronic enteropathy (CE) and a protein losing enteropathy (PLE).^20^, ^21^, ^22^ Despite the associations between vitamin D status and numerous health outcomes in dogs, knowledge about the non‐skeletal effects of vitamin D remain poorly explored in companion animals. Specifically, it remains unclear which canine cell types express VDR and which factors influence the expression of VDR. The aim of this study was therefore to establish which non‐skeletal tissues express the VDR in dogs. We also aimed to investigate how the expression of VDR changes in response to inflammation, using the canine duodenum as our study organ.

## Method

### Healthy Control Dog Population and Sample Procurement

This study was approved by the University of Edinburgh Veterinary Ethical Review Committee. Client consent was obtained for any clinical material to be stored for future research or teaching purposes. Client consent was also obtained for post euthanasia body donation when applicable. Material for non‐skeletal tissue expression of the VDR was collected from 6 healthy dogs that were euthanized for non‐health related reasons. Thirteen non‐skeletal tissues were collected from these 6 dogs; stomach, duodenum, ileum, colon, skin, kidney, spleen, liver, mesenteric lymph node, heart, lung, and either ovary or testicle. All samples from cadavers were collected within 30 minutes of euthanasia to reduce the impact of autolysis. These samples were formalin fixed and paraffin wax‐embedded blocks were made using standard methods.

### Immunohistochemistry

Four micron thick sections were cut and dried overnight at 37°C on Superfrost Ultra Plus slides,^1^ and then at 60°C for 25 minutes. After rehydration with xylene and absolute ethanol the slides were rinsed with tris buffered saline tween (TBST). Antigen retrieval was performed by immersion in 0.01 M citrate acid, pH 6.0, or high pH buffer^b^ (H‐3300) at 110°C for 5 minutes (12 minutes total heat time). Slides were then cooled for 5 minutes in running water. This was followed by rising in TBST. Sections were incubated overnight at 4°C with and without the primary antibody (Table 1), and negative controls were made with an isotype control primary antibody (Table 1). Validation and evaluation of the optimal concentration of each primary antibody was performed using serial antibody dilutions on the respective positive controls. Following incubation with the primary antibody, endogenous peroxidases were blocked with Dako REAL blocking solution (Agilent S2023)^h^ for 10 minutes. The following secondary reagents and times were used: ImmPRESS anti rat HRP mouse (MP‐7444),^2^ 15 minutes at room temperature, Envision anti rabbit HRP (Agilent K4011)^h^ or Envision anti mouse HRP (Agilent K4007),^h^ 40 minutes at room temperature depending on the primary antibody. After each incubation step, the sections were rinsed in TBST 3 times. Visualization was performed using DAB and Chromogen (Agilent K3468)^h^ for 10 minutes. The sections were counterstained with Harris hematoxylin for 20 seconds, dehydrated and mounted using ClearVue mountant.^a^ Immunohistochemistry (IHC) for each antibody was performed in batches of between 15 and 20 and a known positive tissue section was incorporated into each staining run.

**Table 1:** The primary antibodies and isotype controls used in this study.

Antibody	Antibody Name	Antibody Type	Dilution
VDR	Purified Rat IgG2b (9A7)^3^	Monoclonal Rat	1:8000
VDR Isotype	Purified Rat IgG2b Isotype Control (RTK4530)^4^	Isotype Control	1:100
ECAD	Purified Mouse Anti‐E‐Cadherin^5^	Monoclonal Mouse	1:4000
ECAD Isotype	Purified Mouse IgG2a κ Isotype Control^6^	Isotype Control	1:100
CLD2	Rabbit anti‐Claudin‐2^7^	Polyclonal Mouse	1:2000
CLD2 Isotype	Rabbit IgG Isotype Control^8^	Isotype Control	1:100

### Chronic Enteropathy and Control Dog Population and Sample Procurement

For the second aspect of the study, a comparison of dogs with CE and control dogs, 2 populations were enrolled for assessment of duodenum, as this was previously shown to express VDR.^23^ Control dogs for this aspect of the study, were prospectively enrolled between 2013 and 2016 if they were being euthanized for non‐gastrointestinal related reasons and owners had given consent for body donation for research and teaching. Inclusion criteria were as follows: no recent history of gastrointestinal clinical signs, a negative fecal parasite analysis, and no recent medications. Full thickness samples from the duodenum were collected for histology (formalin fixed) and endoscopic biopsy forceps were used to collect samples from the duodenum for gene expression (RNALater).^9^

Client owned dogs with CE were enrolled prospectively over the same period. For inclusion into the study dogs were required to have clinical signs for at least 4 weeks, with all other causes of intestinal inflammation excluded through standard examinations (hematology, biochemistry, fecal analysis, abdominal ultrasound, endoscopy, and histology). For each dog, details were also recorded to determine the canine inflammatory bowel disease activity index (CIBDAI) score.^24^ Dogs were excluded if they had been treated with glucocorticoids within 4 weeks of the procedure. Gastrointestinal endoscopy was performed as standard and biopsy samples were fixed in formalin and submitted for histopathological assessment for diagnostic purposes. At the time of endoscopy, a biopsy sample was also stored in RNALater.^i^ These samples were agitated at 4°C for up to 24 hours and then stored at −80°C until batch RNA extraction. Histopathology of all samples, control, and affected, was reviewed by a single pathologist. Classification and scoring were performed based on criteria previously published by the World Small Animal Veterinary Association (WSAVA).^25^, ^26^ This score consisted of a standard assessment of 4 types of infiltrating leukocytes (intraepithelial lymphocytes, lamina propria lymphocytes, neutrophils, and eosinophils) and 5 morphological features (crypt distention, lacteal dilatation, mucosal fibrosis, villous stunting, and epithelial injury), all scored from 0 to 3. This numerical score was cumulated and a total score allocated to each dog, alongside a categorical morphological diagnosis (normal, mild, moderate, and marked inflammation).

### Quantification of Immunoreactive Cells

All sections were analyzed by the same clinical pathologist (EM), who was blinded to case details. Quantification of VDR immunolabeling was performed by a semi‐quantitive method similar to previously described.^27^

Evaluating the entire section, the overall stain intensity was scored from 0 to 3, with 0 = no stain/weak, 1 = moderate, 2 = strong, and 3 = very strong. The percentage of cells stained was also then evaluated with 0 = none to < 5%, 1 = < 25% of cells, 2 = 25–50% of cells, 3 = 51–75% of cells, 4 = >75% of cells stained. The total score per sample was then calculated by intensity × percentage of cells, giving a maximum range of the overall score (OS) of 0–12.

Duodenal samples from dogs with CE and control dogs were also analyzed by IHC for 2 tight junction elements claudin 2 (CLD2) and E‐Cadherin (ECAD), as outlined in Table 1, which have been reported to change with inflammation.^28^, ^29^

The IHC for each antibody was scored according to the protocol above. To ensure consistency, example images of each intensity score for each antibody were used as a guide throughout the scoring process and all slides were scored twice on different days. To also factor for sample depth, the intestinal samples were allocated an additional separate score for the villous tips, mid‐villous region, and crypts. For forcep biopsy samples, all biopsies on the sample slide were examined and scores assigned for the slide as a whole. For the whole tissue samples from healthy dogs, each tissue was scored for the tissue as a whole and where staining was present in a particular cell type, nuclear, and cytoplasmic staining were recorded as present or absent.

### Total Ribonucleic Acid Extraction and Reverse Transcription

Total RNA for quantitative real‐time polymerase chain reaction (qPCR) analysis was isolated from the duodenal tissue by first homogenizing the tissue with TRIzol.^10^ A single biopsy was added with TRIzol to a lyzing matrix D tube^i^ and agitated at 4 m/s for 20 seconds with a Precellys 24.^j^ The mixture was incubated for 3 minutes with 1‐Bromo‐3‐chloropropane,^11^ followed by centrifugation to elute the RNA. The RNA from the homogenate was then extracted with a QIAGEN RNeasy mini kit^12^ per the manufacturer's protocols. RNA concentrations from all samples were measured on the NanoDrop ND‐1000.^13^ RNA purity was assessed with the (A260/A280) value from the NanoDrop and by an Agilent Tapestation 2100.^14^ Synthesis of complementary deoxyribonucleic acid (cDNA) was performed using the QIAGEN Omniscript Reverse Transcription kit^l^ per the manufacturer's protocols. Briefly, a master mix of deoxynucleotide (dNTP), RNase inhibitor, Omniscript Reverse Transcriptase, RNAse‐free water, a buffer solution and Oligo‐dT primer^15^ was added to each 1,000 μg of template RNA. These solutions were then incubated at 37°C. The product cDNA was stored at −80°C until qPCR reactions were performed in batches.

### Quantitative Real‐Time Polymerase Chain Reaction

Real‐time qPCR experiments were performed in a Roche Light Cycler 480^16^ with a 12 µL reaction volume containing 4.5 µL cDNA, 1.25 µL primers, and 6.25 µL SYBR Green qPCR Master Mix. Reactions were also performed without reverse transcriptase and without template, using distilled deionized H_2_O to maintain volume to monitor for contamination. A standard cycling program was used. Samples were run at 50°C for 2 minutes, 95°C for 2 minutes, and then 40 cycles of 95°C for 15 seconds followed by 60°C for 30 seconds.

Primers for the VDR, 25‐hydroxyvitamin D3 1‐alpha‐hydroxylase (CYP27B1), ECAD, and CLD2 (Table 2) were designed by the Roche primer design software based on canine sequences from the Ensembl database as previously described,^30^ so that the predicted amplicon would span exon‐exon boundaries. The primers were assessed by Basic Local Alignment Search Tool analysis (National Center for Biotechnology Information). Sequences were tested with nucleic acid folding software (OligoAnalyser 3.1) in concordance with MIQE guidelines.^31^ Primer sequences used for glyceraldehyde 3‐phosphate dehydrogenase (GAPDH), actin beta (ACTB), and Succinate dehydrogenase complex subunit A (SDHA) were previously described (Table 2).^32^, ^33^

**Table 2:** Reference and target gene primer sequences and reaction efficiency.

Reference or Target	Gene	Forward Primer (5′3′) Reverse Primer (5′‐3′)	Product Length (bp)	Tm (°C)	Reaction Efficiency	*R* ^2^
Reference	ACTB	CCAGCAAGGATGAAGATCAAG	100	57.9	110	0.98
		TCTGCTGGAAGGTGGACAG		58.8		
Reference	SDHA	GCCTTGGATCTCTTGATGGA	92	56.9	97	0.98
		TTCTTGGCTCTTATGCGATG		55.9		
Reference	GAPDH	TGAAGGGGTCATTGATGGCG	90	60.3	95	0.99
		TCAACGGATTTGGCCGTATTGG		59.4		
Target	VDR	ACTTGCATGAGGAGGAGCA	114	59	98	0.97
		TGTTGGACAGGCGGTCTT		60		
Target	CYP27B1	CTGTATGAACTCGCTCGGCA	159	60	94	0.97
		AGGGTACAGTCTCAGCACCT		59		
Target	CLD2	CAGCCCCTTGCAACTAGAGG	105	60.4	107	0.99
		GCCCCTGGTTCTTCACACAT		60.3		
Target	ECAD	TCAACCCAACCACGTACCAG	87	59.9	98	0.99
		GGACATCAGCATCCGTCACT		59.8		

Specificity of the products was verified for each target and referenced with gel electrophoresis showing a single product of the desired length. In addition, a melting curve analysis was performed for each reference and target.

Multiple reference genes were selected in line with available guidelines.^34^ Reference genes (Table 2) were selected based on previous evidence of high expression within the canine duodenum.^33^ Analysis of the reference genes' relative expression levels by the relative expression software tool (REST) (M. Pfaffl [Technical University Munich] and QIAGEN http://www.gene-quantification.de/rest-2009.html) identified no significant differences among groups (control, CE, and PLE). Reference gene expression was also shown to be stable using Best Keeper software.^35^

To minimize any technical, run‐to‐run variation between different samples for the comparison of gene expression, the maximum number of samples (in triplicate) were analyzed in each run.^34^ As not all samples could be analyzed for 1 gene in the same run, control, and affected cases were spread across 2 runs equally and inter‐run calibrators were included. A correction factor was then generated to control for inter‐run differences.

### Statistical Analysis

Relative expression software tool (REST), which has been previously validated,^31^ was used to analyze the qPCR results. This software incorporates PCR efficiency correction and reference gene normalization. It integrates a statistical analysis randomization algorithm to calculate the statistical difference of variation between 2 groups and a bootstrapping technique which provides 95% confidence interval for expression ratios. The REST software uses a P(H1) test for the statistical analysis that involves a robust random sample reallocation to assess for significance.^36^ qPCR data analysis was also performed using the relative quantification method, delta delta CT (ΔΔCT) method.^37^ The relative quantification data generated were log2 transformed (Log2RQ) for normalization.^38^ These Log2RQ values, IHC scores and signalment details were assessed with a statistical program,^17^ which was also used for graphical representation of data.

Normality of data was tested by a D'argestine Pearson Omnibus normality test. Nonparametric analysis of ordinal categorical data was performed with a Mann–Whitney test or for multiple subsets with a Kruskal–Wallis. For continuous numerical data, a Spearman rank correlation was performed. Several different factors were assessed for a relationship to VDR and CYP27B1 expression; control versus dogs with CE, type of disease (control, CE, and PLE), CIBDAI score, overall pathologist morphological diagnosis category, WSAVA score, ECAD, and CLD2 expression. *P* values and *P*(H1) values < 0.05 were considered significant. For REST analysis, a CIBDAI cut off value of 9 was used as this is consistent with severe clinical signs^24^ and the median WSAVA score of 5 was used. Paired scatter plots and univariate analysis were performed from all Log2RQ data before deciding on any additional analysis.

Images were captured with a BX41 light microscope with DP72 camera attachment and Cell^ D^®^ imaging software.^18^

For graphical representation of continuous data, to ensure all data points could be appreciated, minimal randomized jitter was applied to whole data sets.

## Results

### Vitamin D Receptor Expression in Non‐Skeletal Tissue

**Figure 1:**
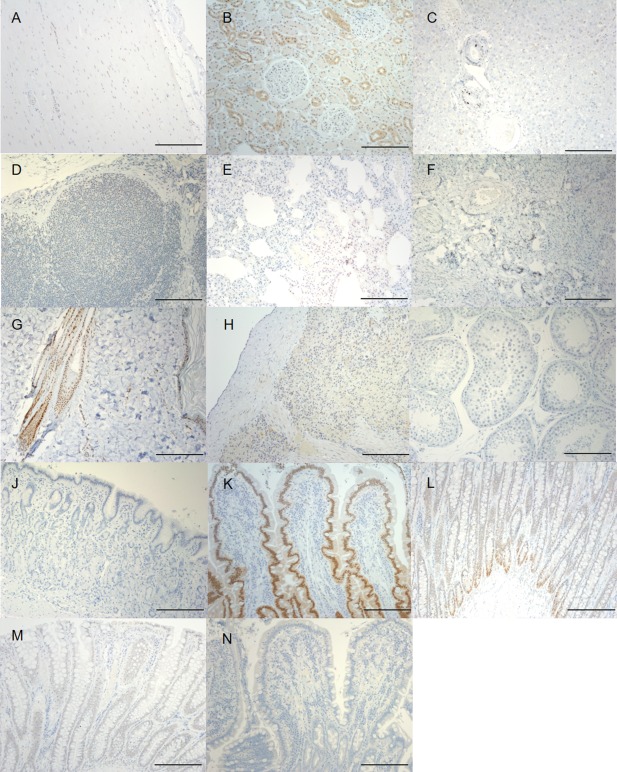
Overall vitamin D receptor immunohistochemistry expression score in the 13 non‐skeletal tissues and an isotype control. Heart **(A)**, kidney **(B)**, liver **(C)**, lymph node **(D)**, lung **(E)**, ovary **(F)**, skin **(G)**, spleen **(H)**, testicle **(I)**, stomach **(J)**, duodenum **(K)**, ileum **(L)** colon **(M)** negative control duodenum, as this organ was shown as it had the highest staining intensity **(N)**. Scale Bar is equal to 200 μm.

The 6 dogs collected for non‐skeletal tissue expression were 2 Staffordshire Bull terriers, a Mastiff, Border Collie, Pitbull terrier, and Jack Russell terrier, with a median age of 4 years (range 2–8 years). Four of the 13 sampled organs were overall moderate to strongly positive for the VDR: duodenum, ileum, kidney, and skin (Fig 1). There was no positive staining in the stomach or testicle samples.

**Figure 2:**
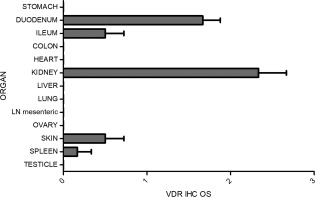
Overall vitamin D receptor immunohistochemistry expression score in the 13 non‐skeletal tissues from 6 dogs.

Of the intestinal sections, duodenum had the highest average IHC OS of 1.67, while ileum was lower at 0.5 OS (Figs 1, 2). In the duodenum, there was strong nuclear labeling of mucosal enterocytes, with weaker cytoplasmic labeling. In both the duodenum and ileum the overall VDR immunolabelling score was higher in the crypt enterocytes compared to the villous tips; goblet cells within the epithelial monolayer were completely negative for VDR. There was minimal labeling in the colon and typically only within the enterocyte nuclei.

In all renal samples, high numbers of cells stained with moderate intensity (average IHC OS 2.33). Vitamin D receptor labeling in the skin was of moderate intensity, mainly concentrating within the hair bulb, outer root sheath, and shaft (0.5 OS) and the non‐keratinized layer of the epidermis (stratum basale). Immunolabelling of the spleen was weak to moderate (average IHC OS of 0.17). There were several organs that labeled weakly and only within a low number of cells, including lymph nodes, heart, liver, ovary, and lung (Figs 1, 2).

### Signalment and Pathological Scores of Control Dogs and Dogs with Chronic Enteropathy

The 34 dogs with CE included 25 different breeds and 2 cross breed dogs. The 24 dogs without clinical signs of CE included 10 different breeds and 4 cross breed dogs (Table 3). Median age was 5 years for both groups (range 2–17 years). Age and sex were not significantly different between the populations, though there was a nonsignificantly higher proportion of male dogs in the CE group.

**Table 3:** Breeds composing the two groups, chronic enteropathy, and control dogs.

CE breeds	Control Breeds
Greyhound	Border Collie × 3
Airedale Terrier	Bull terrier
Alaskan Malamute	Canadian Mastiff × 2
Border Collie × 3	Cross breed × 4
Boxer	Labrador
Cross breed × 2	Pitbull × 2
Cavachon	Rottweiler
Cocker Spaniel	Samoyed
English Springer Spaniel	SBT × 7
Flat Coat Retreiver	Yorkshire Terrier
French Bulldog	WHWT
German Short Haired Pointer Golden Retriever Gos D'Atura Catalan Irish Setter Japanese Akita JRT × 2 Labrador × 3 Lakeland Terrier × 2 Lhasa Apso Lurcher Maltese Terrier Nova Scotia Duck Tolling Pekinese Rottweiler WHWT × 3	

The median CIBDAI of CE dogs was 9 (range 3–16) and the duration of clinical signs before presentation was a median of 7 weeks (range 4 weeks to 3 years). Inflammatory changes were present in all dogs with CE and the median WSAVA score was 5 (range 1–14). The WSAVA score was significantly higher in dogs with CE compared to controls (*P* = 0.0001). Of the 34 dogs with CE, 10 had a low serum albumin and were classified as a PLE.

Of these, 1 control dog was excluded from the IHC analysis because of lack of sufficient sample. One control dog was removed from qPCR analysis because of low RNA quality and finally, 2 CE dogs were removed because of consistently high threshold cycle values indicating sample degradation during the final stages of sample preparation.

### Duodenal Vitamin D Receptor Expression in Control Dogs and Dogs with Chronic Enteropathy

**Figure 3:**
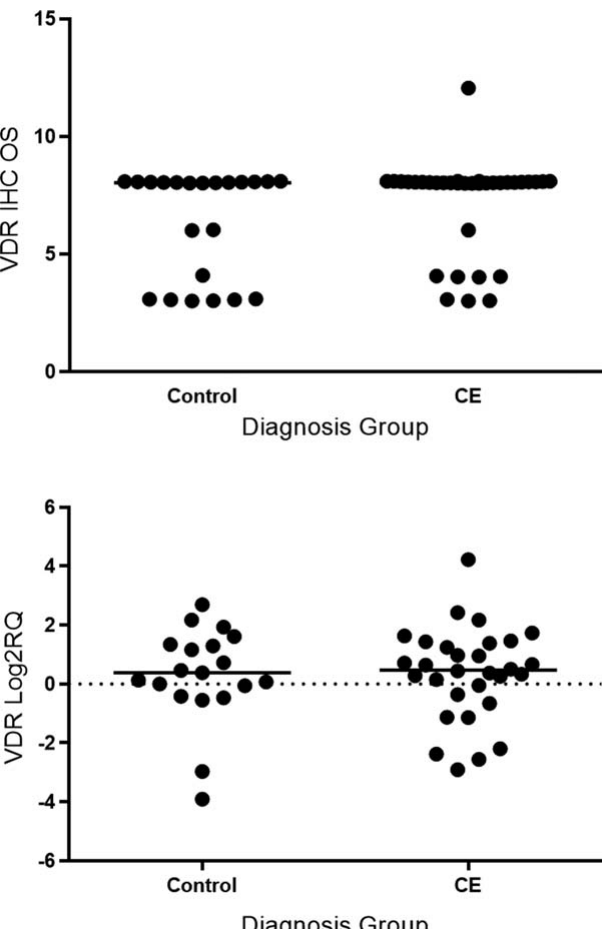
Vitamin D receptor (VDR) immunohistochemistry overall score (left) and VDR Log2RQ value (right) for control and CE dogs.

**Figure 4:**
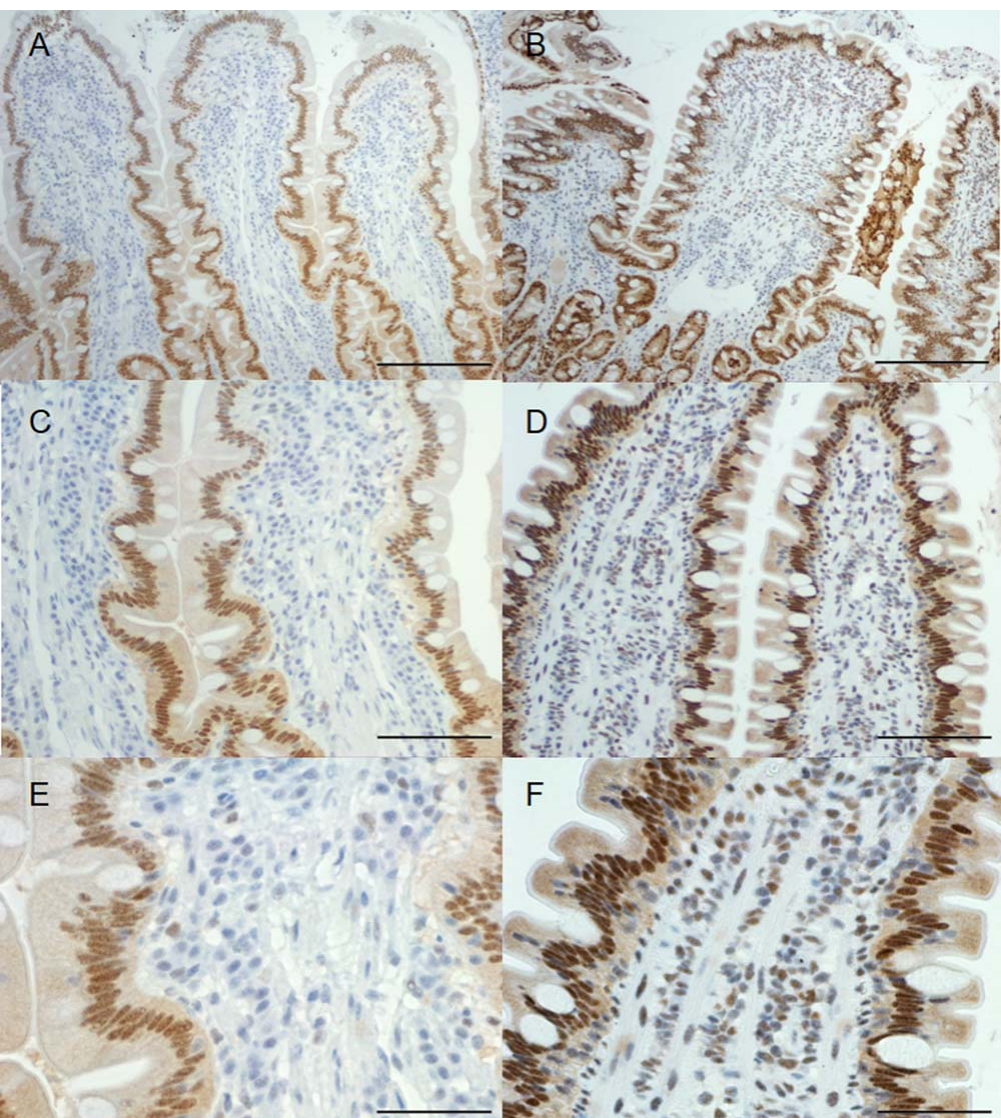
Duodenum of control dogs **(A, C, E)** and dogs with chronic enteropathy **(B, D, F)** illustrating immunolabelling of Vitamin D receptor. Scale Bar is equal to 200, 100, and 50 μm, respectively.

VDR mRNA expression was identified in the duodenum of both control dogs and those with CE. REST analysis identified no significant difference between controls and dogs with CE or PLE. Mann–Whitney statistical testing identified no significant difference in VDR qPCR expression (Log2RQ values) between control dogs and dogs with CE, *P* = 0.87. There was also no significant difference between these populations in protein expression (IHC OS), *P* = 0.099 (Figs 3, 4). RNA expression of CYP27B1 was low for both control dogs and those with CE. REST analysis and Log2RQ value analysis of CYP27B1 identified no significant differences in RNA expression, *P* = 0.22.

### Duodenal Vitamin D Receptor Expression from Dogs with Chronic Enteropathy with Increasing Inflammation

REST analysis showed no significant difference in VDR or CYP27B1 expression between dogs with mild inflammation and dogs with moderate to marked inflammation. There was no significant difference in expression when a high WSAVA score (ie > 5) was compared to a score of 2 or less and no difference in expression when a high CIBDAI score (ie > 9) was compared to a score of <3.

**Figure 5:**
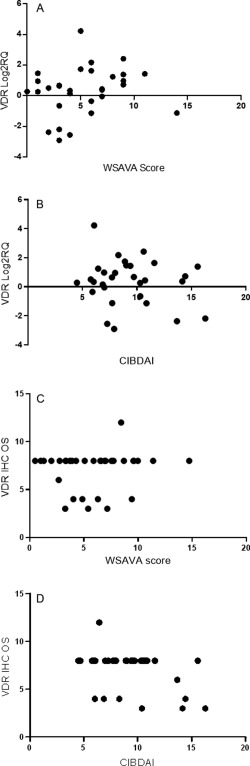
Vitamin D receptor (VDR) immunohistochemistry overall score and VDR Log2RQ value for dogs with chronic enteropathy plotted against histological inflammation (World Small Animal Veterinary Association score) **(A and C)** or severity of clinical signs (canine inflammatory bowel disease activity index) **(B and D)**.

Spearman rank correlation showed no association between VDR RNA expression (Log2RQ), the WSAVA score (*r* = 0.2288, *P* = 0.11) and the CIBDAI (*r* = −0.1488, *P* = 0.39). There was also no correlation between the VDR IHC OS and the WSAVA score (*r* = 0.2204, *P* = 0.099) and CIBDAI, (*r* = 0.05171, *P* = 0.7) (Fig 5). Spearman rank correlation showed no association between CYP27B1 RNA expression (Log2RQ), the WSAVA score (*r* = 0.2165, *P* = 0.3) and the CIBDAI (*r* = −0.1538, *P* = 0.47).

### Comparison of Duodenal Vitamin D Receptor Expression and Related Proteins in Dogs with Chronic Enteropathy

Spearman rank correlation showed no association between VDR RNA expression (Log2RQ) and RNA expression of two of the tight junction components CLD2 and ECAD. There was also no correlation between the respective overall IHC scores for VDR, and RNA expression of CLD2 and ECAD.

## Discussion

This study establishes which non‐skeletal tissues express VDR in the dog. The tissues expressing VDR at the highest level; kidney, duodenum, and ileum were an expected finding based on a previous study in dogs^23^ and the known classical calcium homeostasis roles of these tissues. The moderate expression within the skin has not previously been reported in the dog. There were multiple tissues with weak positive expression of VDR, with only the stomach and testicle being negative. The second major finding of this study was that duodenal VDR expression, both RNA and protein, did not decrease with inflammation. This contrasts with the changes reported in both human and rodent colonic inflammation.^39^, ^40^

Our study is the first to establish that canine skin expresses high levels of VDR, a finding that is similar to those observed in other species.^41^ This finding is noteworthy, given the fact that the dog does not produce significant levels of cutaneous vitamin D from ultraviolet light.^42^, ^43^ In cats, there is also a lack of cutaneous vitamin D production, caused by increased activity of the enzyme 7‐dehydrocholesterol reductase,^44^ but the cause is unknown in the dog. Active vitamin D metabolites stimulate differentiation and inhibit proliferation of human keratinocytes via the VDR.^45^ The expression, particularly within the hair bulb, has also been documented in people and in mouse models. VDR mouse knockout models develop alopecia, indicating that the VDR is important for follicular growth.^41^, ^46^ It is intriguing to speculate whether there are breed differences with regards to VDR receptors in the skin; especially with reference to the hairless Chinese Crested dog.

Weakly positive VDR expression in the lymph node and spleen is consistent with findings in people^1^, ^41^ and mice.^47^, ^48^ Specifically, VDR has been identified in activated human inflammatory cells^2^ and 1,25(OH)_2_D has been shown to inhibit T cell proliferation.^49^ VDR has also been immunohistochemically identified in neoplastic canine mast cells.^50^ Some of the positive cells in the lungs, lymph nodes, spleen, and liver could be mast cells or other inflammatory cells. There was only weak VDR expression in the colon in healthy dogs, a finding which is different to people and other species.^39^, ^51^ In human ulcerative colitis and in mouse models of colitis, VDR expression is negatively correlated with colonic inflammation.

In contrast to findings in humans with IBD, which have significantly decreased intestinal VDR expression,^39^, ^52^ we found no difference in VDR expression in the duodenum of dogs with gastrointestinal inflammation. Assessments of inflammation by several criteria and by multiple methods showed no significant decline in VDR (Fig 4). There was also no correlation identified with ECAD, a tight junction element, which has been shown to decrease with ulcerative colitis and Crohn's disaese^53^, ^54^ and IBD in dogs.^28^ Last, there was no correlation with CLD2, another tight junctional element that increases with intestinal inflammation in people,^55^, ^56^, ^57^ and in dogs with CE.^29^

There are numerous studies which indicate a role for the VDR in the protection against inflammation. For example, VDR null mice develop more severe colitis, and clinical signs can be attenuated by reconstitution of the intestinal epithelial VDR.^58^ Vitamin D and the VDR have been shown to be important regulators of the immune system in IBD.^59^ It has also been clearly demonstrated that serum vitamin D is reduced in dogs with CE^20^ and is negatively correlated with inflammation.^22^ In several cases of canine CE there is a documented decrease in calcium^21^ and increased parathyroid hormone (PTH).^20^ The VDR and 1,25(OH)_2_D are both required for calcium absorption, so in this species, despite high expression of the VDR, there is evidence that calcium becomes low enough to result in an increase in PTH, because of lack of 1,25(OH)_2_D.

A functional local vitamin D synthesizing system has been indicated as important for the prevention of IBD.^60^ The fact that the VDR is highly expressed in dogs regardless of inflammation could indicate that a lack of the binding substrate, 1,25(OH)_2_D is more important in the pathogenesis of the inflammation than the receptor itself. This could be caused by systemically low serum 25(OH)D, as has been measured,^20^ or because of low local activity levels of CYP27B1 or, finally, because of increased 1,25‐dihydroxyvitamin D_3_ 24‐hydroxylase, (CYP24A1) levels, which normally reduce 1,25(OH)_2_D levels in a negative feed‐back manner. It is likely that low serum 25(OH)D occurs in CE because of intestinal loss of Vitamin D and its metabolites, which are bound to plasma vitamin D binding protein, as previously suggested.^61^ It would therefore be plausible to investigate the effects of supplementation of calcitriol (1,25(OH)_2_D) in dogs with CE that have low serum 25(OH)D. This concept is further supported by previous studies showing that 1,25(OH)_2_D or a vitamin D analogue, TX527, ameliorated inflammation, and clinical signs in spontaneous murine models of IBD.^62^, ^63^ A protective effect of 1,25(OH)_2_D has also been reported in mouse models of hepatitis^64^ and there are several studies indicating improved immune tolerance,^65^ decreased proinflammatory cytokines^66^ and increased anti‐inflammatory cytokine responses with 1,25(OH)_2_D.^67^, ^68^

A lack of significant change in qPCR expression of CYP27B1 would indicate that this is not an enzyme that is affected in dogs with CE, as has been observed by some studies of human IBD^69^ and intestinal neoplasia.^70^ This would further support a potential beneficial outcome of the addition of calcitriol in the treatment of canine CE, with less requirement to consider the hydroxylation steps, as some authors have sought to do.^71^ The expression of CYP27B1, however, in this study was overall very low.

Our study confirms the expression of VDR in a large number of non‐skeletal tissues in the dog. We also showed that intestinal VDR expression does not decrease in the presence of inflammation, in contrast to humans and rodents. The mechanism for this difference is unknown, but it could reflect a more profound effect of systemically low serum 25(OH)D on the development of CE. One potential clinical implication is consideration of 1,25(OH)_2_D or its analogues to ameliorate clinical signs. In this species, as the VDR is still expressed at high levels in the duodenum, there is higher available binding potential for ligand, which is more likely to translate to a beneficial effect. Alongside this, as vitamin D concentrations are less affected by sunlight in the dog, there are fewer external environmental factors to consider, which may increase the dog's value as a naturally occurring model of the interplay between vitamin D and chronic inflammatory conditions.

## Footnotes

1 Thermo Fisher Scientific, Saarbruckener, Braushweig, Germany

2 Vector Labs, Burlingame, CA

3 Thermo Fischer Scientific, Rockford, IL

4 BioLegend, London, UK

5 BD Biosciences Transduction Laboratories, Oxford, United Kingdom

6 BD Biosciences Pharmingen, Oxford, United Kingdom

7 Invitrogen Corporation, Camarillo, CA

8 Dako North America Inc, Carpnteria, CA

9 Ambion life technologies, Carlsbad, CA

10 Bertin Technologies, Montigny‐le‐Bretonneur, France

11 Sigma‐Aldrich, St Louis, MO

12 QIAGEN GmbH, Hilden, Germany

13 Spectrophotometer, Version 3.7.1, Thermofisher Scientific, Wilmington, DE

14 Agilent Technologies Inc, Santa Clara, CA

15 PROMEGA, Madison, WI

16 Roche‐Applied‐Science, 05015243001, Manheim, Germany

17 Graph Pad Prism Version 6.05, GraphPad Software, San Diego, CA

18 Olympus Ltd, Southend‐on‐Sea, United Kingdom
